# Functional polymorphisms in the TERT promoter are associated with risk of serious ovarian and breast cancer

**DOI:** 10.1186/1897-4287-10-S2-A86

**Published:** 2012-04-12

**Authors:** Jonathan Beesley, Hilda Pickett, Sharon Johnatty, Xiaoqing Chen, Jun Jun Li, David Rider, Michael Stutz, Diether Lambrecht, Jenny Chang-Claude, Thilo Dork, Marc Goodman, Bart Kiemmney, Elisa Bandera, Linda Kelemen, Shan Wang-Gorke, Ian Campbell, Simon Gayther, Susan Ramus, Ellen Goode, Roger Reddel, Georgia Chenevix-Trench

**Affiliations:** 1 Division of Genetics and Population Health, Queensland Institute of Medical Research, Brisbane, Queensland, Australia; 2 Cancer Research Unit, Children's Medical Research Institute, Westmead, New South Wales, Australia; 3 Sydney Medical School, University of Sydney, Sydney, New South Wales, Australia; 4 Department of Health Sciences Research, Mayo Clinic College of Medicine, Rochester, Minnesota, USA; 5 Vesalius Research Center, VIB, Leuven, Belgium; 6 Vesalius Research Center, University of Leuven, Leuven, Belgium; 7 Division of Cancer Epidemiology, German Cancer Research Center (DKFZ), Heidelberg, Germany; 8 Department of Obstetrics and Gynecology, University of Ulm, Ulm, Germany; 9 Clinics of Obstetrics and Gynaecology, Hannover Medical School, Hannover, Germany; 10 Cancer Epidemiology Program, University of Hawaii Cancer Center, Honolulu, Hawaii, USA; 11 Department of Epidemiology, Biostatistics and HTA, Radboud University Nijmegen Medical Centre, Nijmegen, The Netherlands; 12 Department of Urology, Radboud University Nijmegen Medical Centre, Nijmegen, The Netherlands; 13 Comprehensive Cancer Center, The Netherlands, Nijmegen, The Netherlands; 14 The Cancer Institute of New Jersey/Robert Wood Johnson Medical School, New Brunswick, New Jersey, USA; 15 Department of Population Health Research, Alberta Health Services-Cancer Care, Calgary, Alberta, Canada; 16 Peter MacCallum Cancer Centre, East Melbourne, Victoria, Australia; 17 Department of Preventive Medicine, Keck School of Medicine, University of Southern California, Los Angeles, California, USA

## 

Genome-wide association studies have implicated the *TERT-CLPTM1L* locus at 5p15.33 in susceptibility to a variety of cancers including pancreas, lung, skin and glioma, suggesting that *TERT* may act as a “pan-cancer susceptibility locus” in a similar manner to the 8q24 region.

We initially identified an association between an intronic *TERT* SNP rs7726159 and epithelial ovarian cancer (EOC) risk through an Illumina GoldenGate scan of single nucleotide polymorphisms (SNPs) in candidate genes, with a more pronounced effect in serous cases (Johnatty et al, PloS Genetics 2010). We employed a fine-mapping strategy of a 500 kb region of the *TERT-CLPTM1L* locus using data from the 1000Genomes and HapMap Projects, and cases and controls from the Ovarian Cancer Association Consortium (OCAC). Using single-marker and stepwise logistic regression adjusted for age and study, we analysed 28 SNPs in 2,130 invasive epithelial ovarian cancer cases, including 1,076 of serous histology, and 3,975 controls of Caucasian ancestry from nine OCAC studies, and observed a significant association between serous cases and a *TERT* promoter SNP rs2736109 [adj. OR_per-allele_ 0.86 (0.77-0.96), *P* = 0.005].

Since much of the genetic architecture is shared between EOC and breast cancer, we analysed rs2736109 in 4,277 invasive breast cancer cases and 7,000 controls from the Breast Cancer Association Consortium (BCAC). Although there was no association with invasive breast cancer risk overall [adj. OR_per-allele_ = 0.95 (0.90 - 1.01) *P* = 0.10], we found the strongest evidence of association among ER-negative cases over the age of 50 (n=636) [adj. OR_per-allele_ 0.84 (0.75-0.95), *P* = 0.005].

To examine the potential functional consequences of rs2736109 and another promoter SNP, rs2736108, we generated luciferase reporter constructs comprising 3.7 kb of the TERT promoter containing various combinations of alleles and transfected them into breast and ovarian cell lines. We observed a decrease in luciferase expression by the presence of both the A alleles at rs2736108 and rs2736109, but not when either allele is present alone. Our analysis of 345 Australian controls suggests that the A-A haplotype at rs2736108 and rs2736109 occurs with a frequency of 32%, suggesting that this relatively common promoter haplotype may lower the risk of serous epithelial ovarian cancer though decreasing TERT expression.

